# Short-Term Complete Submergence of Rice at the Tillering Stage Increases Yield

**DOI:** 10.1371/journal.pone.0127982

**Published:** 2015-05-22

**Authors:** Yajie Zhang, Zhensheng Wang, Lei Li, Qun Zhou, Yao Xiao, Xing Wei, Mingyao Zhou

**Affiliations:** 1 Jiangsu Key Laboratory of Crop Genetics and Physiology/Co-Innovation Center for Modern Production Technology of Grain Crops, Yangzhou University, Yangzhou 225009, China; 2 School of Hydraulic, Energy and Power Engineering, Yangzhou University, Yangzhou 225009, China; Meiji University, JAPAN

## Abstract

Flooding is a major threat to agricultural production. Most studies have focused on the lower water storage limit in rice fields, whereas few studies have examined the upper water storage limit. This study aimed to explore the effect of waterlogging at the rice tillering stage on rice growth and yield. The early-ripening late japonica variety Yangjing 4227 was selected for this study. The treatments included different submergence depths (submergence depth/plant height: 1/2 (waist submergence), 2/3 (neck submergence), and 1/1 (complete submergence)) and durations (1, 3, and 5 d). The control group was treated with the conventional alternation of drying and wetting. The effects of waterlogging at the tillering stage on root characteristics, dry matter production, nitrogen and phosphorus accumulation, yield, yield components, and 1-aminocyclopropane-1-carboxylic acid synthase (ACS) gene expression were explored. Compared with the control group, the 1/1 group showed significant increases in yield, seed-setting rate, photosynthetically efficient leaf area, and OS-ACS3 gene expression after 1 d of submergence. The grain number per panicle, dry weight of the aboveground and belowground parts, and number of adventitious roots also increased. Correlation analysis revealed a significant positive correlation between the panicle number and nitrogen content; however, no significant correlation was found for phosphorus content. If a decrease in rice yield of less than 10% is acceptable, half, 2/3, and complete submergence of the plants can be performed at the tillering stage for 1-3 d; this treatment will increase the space available for rice field water management/control and will improve rainfall resource utilization.

## Introduction

Flooding is a major threat to agricultural production. China is one of the largest agricultural production areas in the world, and large areas of the country are devoted to rice cultivation. However, rice planting areas in China are primarily located in the southern region, which is characterized by heavy rainfall, humid conditions, and frequent flooding, as well as in northern low-lying swamp zones. Flooding has thus become a major threat to rice production, and the annual loss in China caused by flooding surpasses tens of billions of yuan [1–4]. Therefore, studies examining the influence of flooding on rice are of particular significance for this country.

The life cycle of rice can be roughly divided into seedling, tillering, jointing and booting, heading-flowering, milk, and maturation stages, and the ability of rice to endure waterlogging varies according to the growth stage. In general, waterlogging in the booting and heading-flowering stages causes more serious yield loss than that in the other stages [5]. Flooding frequently occurs in China from June to August, which is also the period when intermediate-late rice is in the early-middle stage. Numerous studies have examined the influence of flooding on rice tillering [5–7]. Rice plants in the tillering stage possess sufficient carbohydrates for growth; compared with controls, plants in the late tillering stage treated with 2 d of submergence did not show a significant difference in the indices investigated, except for an increase in the vitality of the root system. Rice plants in the tillering stage have higher submergence tolerance than those at other stages, and submergence at this stage has little influence on rice yield; thus, it may be possible to deepen post-rainfall water storage containers and expand the water content control space in paddy fields [5–7]. However, previous studies have only focused on treatment after complete submergence, whereas systematic studies on different severity levels of flooding, such as waist submergence (1/2), neck submergence (2/3), and complete submergence (1/1), have not been reported [8]. According to the literature, rice submergence can result in yellow leaf discoloration, a decrease in the number of green leaves and white roots, a decrease in root absorption, impaired growth and development, and a decrease in lodging tolerance, which can result in underproduction or even a complete lack of yield [9–12]. However, most of these investigations lack systematization and quantification.

The minimum soil water content has been reported in numerous studies exploring water-saving rice irrigation theories. Water-saving irrigation of paddy fields in China can be summarized as four patterns: a combination of shallow water/exposed substrate (in the sun), intermittent flooding, semi-dry cultivation, and rainfall storage [13]. The minimum irrigation control limit of the soil water potential is -30 kPa [14]. The lower water-saving and high-yielding irrigation limit for regeneration after transplantation at the critical leaf stage to ensure effective tillering is -15 to -20 kPa; to ensure effective tillering, the limit is -20 to -40 kPa from the critical leaf stage to the secondary branch differentiation stage [15]. However, intensive studies on the permissible upper limit for water storage are rare [16, 17], particularly with respect to rainwater storage, and as a consequence, rainwater cannot be sufficiently utilized [18–20]. An increase in the upper water storage limit indicates that rice plants must undergo waterlogging stress within a certain time period. Therefore, it is necessary to further investigate the submergence tolerance of rice at different growth stages in areas with abundant rainwater to propose upper indices of drainage control for rice at different growth stages.

C_2_H_4_ (ethylene) is a plant hormone thought to be related to the stress response, and it plays a vital role in modifying the plant’s morphology, physiology, biochemistry, and gene expression to allow the plant to withstand adverse conditions. 1-Aminocyclopropane-1-carboxylic acid synthase (ACS) is the key rate-limiting enzyme involved in catalyzing the synthesis of 1-aminocyclopropane-1-carboxylic acid (ACC) from S-adenosyl methionine (SAM) during ethylene biosynthesis; thus, its activity within plant tissue determines the rate of ethylene generation. Therefore, an examination of ACS gene expression can provide information about the plant’s ability to resist adverse circumstances. The ACS gene is one member of a multigene family, and the expression of each member is regulated at both the transcriptional and translational levels by different stress factors, with complex regulatory pathways. There are six ACS genes in rice: OS-ACS1, OS-ACS2, OS-ACS3, OS-ACS4, OS-ACS5 and OS-ACS6 [21–23]. Specific stress conditions trigger the expression of certain ACS genes, and the enzyme encoded by OS-ACS3 is only expressed in roots [21].

We conducted a study on the submergence tolerance of rice plants at the tillering stage by examining different submergence depths and durations. The aims of this study were to observe changes in the morphological, physiological, and economic traits of tillering rice plants after submergence to explore the underlying causes of these changes from the perspective of modern molecular biology and to determine the critical values of submergence depth and duration for tillering rice.

## Materials and Methods

### Materials and experimental design

This study was conducted at the pot culture testing field of the Institute of High-Efficiency Utilization and Management of Water Resources of Yangzhou University, China, between 2012 and 2013. The test rice variety was early-ripening late japonica (*Oryza sativa L*) Yangjing 4227. Red plastic pots (25 cm × 30 cm) were used. Each pot was filled with 18 kg of well-mixed, sifted surface soil (sandy loam), which was obtained from the preceding wheat field. The major parameters of the soil were as follows [24, 25]: organic matter content, 21.3 g·kg^-1^; effective nitrogen, 101.4 mg·kg^-1^; rapidly available phosphorus, 23.8 mg·kg^-1^; and rapidly available potassium, 59.5 mg·kg^-1^. The seeding was performed on May 14–16 and transplantation on June 14–16 using 3 holes per pot and 2 seedlings per hole.

Flooding was simulated artificially during the tillering stage (15 d after transplantation). The submergence depths (water depth/plant height) were 1/2 (waist submergence), 2/3 (neck submergence), and 1/1 (complete submergence), and the durations were 1, 3, and 5 d. The control group was managed using the conventional water saving method, namely, alternation of drying and wetting. Ten pots were included in each group. The plants were fertilized according to a basal fertilizer/tillering fertilizer/earing fertilizer ratio of 5:2:3. Before transplantation, calcium superphosphate and potassium chloride were applied basally with respective application volumes of 10 g and 5.5 g per pot. The water used for flooding was static, clean tap water. No water changes were conducted during the treatments. After the flooding treatment (water temperature of approximately 30°C), the plants were placed in the shade for 1 d of growth recovery and subsequently moved to natural conditions.

### Determination methods

#### Dynamic growth

Nine seedlings, on which the second main-stem leaf from the top was labeled, were equally transplanted into three pots. Each labeled seedling was transplanted along with an unlabeled seedling into a hole; there were three holes per pot. Leaf age and tiller number were observed every 7 d after post-transplantation regeneration. For each treatment, three pots were observed (each hole served as a unit) to evaluate the critical leaf age at which the tillering stage, the late tillering stage, the shooting stage, the booting stage, the heading-flowering stage, and dynamic tillering occurred.

#### Yield and yield composition

Decreases in the effective panicle number, seed set propagation coefficient, and 1000-seed weight are the major deficiencies caused by waterlogging [26–28]. Three representative pots containing plants in the maturation stage were selected; the effective panicle number and number of high-tillering panicles (sprouting of side stalks from high-node axillary buds of the rice plant’s stem under appropriate conditions is referred to as “high tillering”) were counted, and the spikes were threshed manually. Perfect and imperfect grains were differentiated using the floating method (submerged grains were perfect grains) and recorded. The total grain number, grain number per spike, and seed-setting rate were obtained. The perfect grains were dried in the sun, and 500 grains were weighed to obtain the 1000-seed weight (measurements were performed in triplicate for each treatment). The yield per pot was calculated based on the spike number per pot, grain number per spike, seed setting rate, and 1000-seed weight.

#### High-efficiency leaf area in the heading stage

The three leaves on the upper stalk (flag, top second, and top third leaves) are physiologically young and intercept the most light; therefore, they have strong photosynthetic function and constitute the main functional leaves during the grain filling stage. The total area of these leaves is referred to as the high-efficiency leaf area of the plant. For each treatment, ten representative sample stems in the full heading stage were selected; the total leaf area of the three efficient leaves was determined using an YMJ-B portable leaf area meter (Huier, Hangzhou, China).

#### Photosynthetic rate in the full heading state

The experiment was conducted at 9:00 am on a clear and windless morning. For each treatment group, 6 plants were randomly selected. The net photosynthetic rate of the flag leaves was determined using a portable photosynthesis meter (LI-6400; Li-Cor, Inc., USA) with the following parameters: CO_2_ concentration in the leaf chamber, 380 μmol mol^-1^; light source, red and blue; light quantum flux density, 1400 μmol m^-2^ s^-1^; temperature, 28–30°C.

#### Root morphology and dry weight

In each treatment group, two representative pots were selected at 7 d after post-waterlogging growth recovery. The plants in each pot were placed into a 70-mesh bag. After rinsing, their roots were washed clean with an agricultural compression sprayer. The aboveground parts were removed, and the root system was carefully separated. Using each hole as a unit, a small amount of root material was cut, flash-frozen in liquid nitrogen, and stored at -80°C. This tissue was later used for quantitative expression analysis of the OS-ACS3 gene. The root number per hole was counted for a total of 6 holes. The length of the longest root in each hole was also measured. The thickness of the adventitious roots was measured with a slide gauge as follows: 10 adventitious roots from each hole constituted a group and were placed side by side and subsequently measured; six groups were measured for each treatment. The roots in each hole were packaged and dried at 80°C until a constant weight was achieved. Next, the dry weight was obtained. The aboveground parts were packaged and placed at 105°C for 30 min followed by incubation at 80°C until a constant dry weight was achieved. The dried aboveground parts were kept for further determination of nitrogen and phosphorus.

#### ACS gene expression

Total RNA was extracted from roots with the TransZol reagent (Invitrogen, Carlsbad, CA, USA). DNase I (Invitrogen, Carlsbad, CA, USA) was added to eliminate contaminating genomic DNA. The RNA was reverse transcribed into cDNA with the iScript cDNA synthesis kit (Invitrogen, Carlsbad, CA, USA). Using this cDNA as a template, the expression of OS-ACS3 was determined by real-time polymerase chain reaction (qPCR). The PCR reaction conditions were as follows: 95°C for 30 s, followed by 40 cycles of 94°C for 10 s, 25°C for 25 s and 72°C for 30 s, with a final step of 72°C for 7 min. The experiment was repeated three times. Negative controls were set up with ddH_2_O and RNA in place of the cDNA template to determine whether exogenous DNA or genomic DNA contamination was present.

The primers used in the experiments were designed using Primer Express Software (Foster City, CA, USA) and synthetized by Shanghai Bio Asia Biotechnology Co., Ltd. The purity of the primers was greater than 99%, and Actin was used as the reference gene. The forward primer designed to amplify OsActin (NCBI GenBank: NM_001058705) was 5’-CCAAGGCCAATCGTGAGAAGA-3’, and the reverse primer was 5’- AATCAGTGAGATCACGCCCAG-3’. The primer’s specificity was determined using BL0AST and melting curve analysis. The forward primer designed to amplify OS-ACS3 (NCBI GenBank: AC135427) was 5’-AGTGTGGGAGAGGGTGTGAC-3’, and the reverse primer was 5’-GGTGGAAGTGCCAACAAAG-3’. The expected product size was 214 bp.

Tenfold dilutions of cDNA templates from plants treated with submergence were used for gradient PCR of OS-ACS3 and Actin to obtain standard curves and determine amplification efficiencies. Relative quantitative analysis of each sample was performed according to the literature [29].

#### Plant height

At 10:00 am on the second day after the waterlogging treatment, the height of the main stems (the distance from the ground to the pointed end of the highest leaf) in the experimental and control groups was measured with a ruler. For each treatment, 10 values were obtained to evaluate the influence of waterlogging on rice height.

#### Nitrogen and phosphorus content

The dried aboveground parts were ground and digested with concentrated H_2_SO_4_-H_2_O_2_. The total nitrogen content was determined using the Kjeldahl method [24], and the total phosphorus content was determined using the Mo-Sb colorimetric method [25]. The experiments were performed in triplicate for each treatment. The effect of waterlogging on the nitrogen and phosphorus content in the plants was evaluated.

### Statistical analysis

All data were processed with Excel, and an analysis of variance (ANOVA) was performed using SPSS software (SPSS Inc., Chicago, IL, USA). *T*-tests were employed for comparisons between groups. Charts were drawn with SigmaPlot. Differences at P ≤ 0.05 were considered statistically significant.

## Results and Discussion

In this study, we observed changes in the morphological, physiological, and economic traits of rice plants after submergence at the tillering stage, and we determined the critical submergence depth and duration. Because the results obtained during the two years of this investigation showed similar trends, we focused on the data obtained in 2013.

### Effect of submergence on yield and yield components

#### Yield

The effect of waterlogging on yield is summarized in Table 1. The yields in all treatment groups were significantly decreased compared with that of the control group, except for the 1-d 1/1 submergence group, which showed a significant increase in yield (7.9%). Wang et al. investigated the influence of slight submergence (2 and 4 d) on mid-season rice at the final phase of the tilling stage and found that the yields of the experimental groups were close to those of the control group [7]. Li et al. only sporadically observed yield increases after waterlogging: After neck submergence, rice shows only minor underproduction, and either no underproduction or a production increase is observed when the temperature of the flooded water is close to the optimal temperature range of rice [8]. The differences between our results and the results reported in the literature [7, 8] may be due to differences in the rice waterlogging duration, the waterlogging temperature, the stage of the rice during waterlogging, and the cultivar tested. Our findings indicate that short-term (1 d) complete submergence significantly increased rice yields. This result was presumably associated with a compensatory or super-compensatory effect in the plant upon removal of the waterlogging stress. Super-compensatory effects are a universal phenomenon in biology and are normally caused by stress and damage as a self-accommodation behavior in response to harmful environments. For some crops, the compensatory effects caused by moderate stress are even better than the effects of normal growth conditions, resulting in increased production or minor underproduction [30]. Furthermore, water deficit does not always lead to crop underproduction; indeed, deficit in early stages may lead to production increases in some crops [31].

**Table 1:** Effects of submergence on rice yield, yield components, and effective leaf area in plants at the tillering stage.

Submergence depth-duration (d)	Panicle number per pot	Grain number per panicle	Seed-setting rate (%)	1000-seed weight (g)	Yield (g/pot)	High-tilling panicles per pot	Photosynthetic rate (μmol·cm^- 2^·s^- 1^)	Photosynthetically effective leaf area (cm^2^/ stem)
CK	27.1 bc	105.6 a	91.1 bc	32.5 a	84.6 b	0.0 d	27.9 ab	85.1 b
1/2-1	26.8 bc	99.1 b	93.4 a	32.1 a	79.5 c	0.0 d	27.2 b	77.0 c
1/2-3	27.5 bc	98.4 b	88.9 de	32.0 a	77.0 d	0.3 cd	26.5 bc	74.7 cd
1/2-5	27.3 bc	99.2 b	86.3 f	32.2 a	75.2 d	1.3 bc	26.1 bc	73.7 cd
2/3-1	26.3 c	98.0 b	92.8 ab	32.1 a	76.8 d	0.3 cd	27.7 ab	87.8 ab
2/3-3	27.2 bc	94.3 c	90.3 cd	32.5 a	75.3 d	0.5 cd	25.4 c	74.6 cd
2/3-5	27.8 bc	93.2 c	88.1 ef	31.7 a	72.3 e	1.7 b	25.0 c	73.3 cd
1/1-1	28.2 b	105.8 a	93.4 a	32.8 a	91.3 a	0.5 cd	28.6 a	89.6 a
1/1-3	31.8 a	88.0 d	88.0 ef	32.1 a	79.1 c	1.2 bc	24.8 c	72.7 cd
1/1-5	32.0 a	87.5 d	87.7 ef	31.3 a	76.9 d	2.8 a	23.4 d	71.4 d

Values followed by a different letter are significantly different (*t*-test, P ≤ 0.05).

#### Yield components

As shown in Table 1, compared with the control group, the panicle number in the 1/1 group increased by 4.2% after 1 d of submergence, with significant increases observed after 3 and 5 d of submergence (17.5% and 18.2%, respectively). Further observations revealed that the majority of these panicles were elevated tillering panicles: the deeper the submergence water and the longer the submergence duration, the greater the increase in the elevated tillering panicle number per pot (Table 1).

Table 1 also shows that except for the 1-d 1/1 group, which exhibited a significantly increased seed-setting rate (2.5%) (no significant differences in the grain numbers per panicle or the 1000-seed weight were observed in this group), the other groups showed an increasing panicle number and decreasing grain number per panicle and seed-setting rate as the submergence duration and depth increased. No significant differences in the 1000-seed weight were observed among the different treatment groups.

Our findings regarding the grain number per panicle and seed-setting rate are mostly consistent with those reported in the literature [5–7, 9]. However, the panicle number was different from that reported previously [5]. This is likely because other studies did not take effective elevated tillering panicles into account; were those panicles considered, the panicle number would have increased, though with a smaller panicle size. During waterlogging, the photosynthetic intensity and photosynthetic area of rice decrease, which leads to decreased photosynthetic productivity, insufficient assimilate production, decreased root function and lodging resistance ability due to insufficient oxygen, and inconsistent differentiation and development of young ears. In addition, decreases in the glumous flower number per panicle, seed-setting rate, and 1000-seed weight are observed. Consequently, decreased production or even no production occurs [26–28]. In this study, the 1/1 group showed a significant increase in the seed-setting rate as well as increases in panicle number, grain number per panicle, and 1000-seed weight compared with the control group after 1 d of submergence, which was the primary reason for the significant yield increase in the 1-d treatment 1/1 group. The underlying reason is presumably as follows: compared with partial submergence, complete submergence stress for a short duration (e.g., 1 d) more strongly stimulates photosynthesis in rice leaves, increases the photosynthetic area, promotes the production and accumulation of photosynthate, and enhances grain filling; thus, the plant displays a super-compensatory effect [30, 31]. However, as the submergence duration increases, such compensatory effects may decrease; for example, rice plants do not show an increase in yield after 2 d of complete submergence [7, 8], though an insignificant difference has been reported in the literature [8].

#### Photosynthetically efficient leaf area and photosynthetic rate at the heading stage

As shown in Table 1, the photosynthetically efficient leaf areas of the 1/2 group significantly decreased by 9.5%, 12.2%, and 13.4% after 1, 3, and 5 d of submergence, respectively, compared with those of the control group. The photosynthetic rates did not decrease significantly. The 2/3 group showed significant decreases in efficient leaf area of 12.3% and 13.9% after 3 and 5 d of submergence, respectively. The photosynthetic rate did not decrease significantly after 1 d of submergence and decreased significantly by 8.7% and 10.3% after 3 and 5 d of submergence, respectively. The 1/1 group exhibited a significant increase of 5.3% and significant decreases of 14.6% and 16.1% after 1, 3, and 5 d of submergence, respectively. The photosynthetic rate increased by 2.6% after 1 d of submergence and significantly decreased by 11.0% and 16.1% after 3 and 5 d of submergence, respectively. These results indicate that exposure to the 1/1 treatment for 1 d significantly increased the photosynthetically efficient leaf area of rice plants, whereas both the 3-d and 5-d treatments significantly decreased the photosynthetically efficient leaf area and photosynthetic rate. Increases in the photosynthetically efficient leaf area and photosynthetic rate of rice plants at the heading stage after 1 d of complete submergence have not been previously reported in the literature.

### Effects of submergence during rice tillering on the aboveground dry weight and root system

The effects of submergence during rice tillering on the aboveground dry weight and related roots are summarized in Table 2.

**Table 2:** Effects of submergence on the aboveground dry weight and root characteristics of rice plants at the tillering stage after 7 d of recovery.

Submergence depth-duration (d)	Aboveground dry weight (g/hole)	Adventitious roots (number/ hole)	Dry root weight (g/hole)	Length of the longest root (cm)	Root width (mm/bar)	Relative expression of the OS-ACS3 gene
Control-1	10.42 bc	387 a	1.661 ab	23.5 ab	0.973 bc	1.7 h
Control-3	11.34 ab	385 a	1.727 ab	24.1 ab	0.967 bc	1.6 h
Control-5	12.82 a	388 a	1.704 ab	24.4 a	0.987 abc	1.6 h
1/2-1	11.21 ab	357 ab	1.792 a	24.0 ab	0.960 c	11.4 e
1/2-3	10.81 ab	348 ab	1.688 ab	23.2 abc	0.947 c	6.3 f
1/2-5	10.55 abc	339 a	1.587 ab	22.0 bcd	1.003 abc	3.6 gh
2/3-1	10.11 bc	320 b	1.598 ab	22.0 bcd	0.953 c	16.6 d
2/3-3	9.68 bcd	326 b	1.334 abc	21.3 cde	1.033 ab	10.0 e
2/3-5	8.30 cd	321 b	1.243 bc	20.2 de	1.047 a	5.9 fg
1/1-1	10.50 abc	399 a	1.685 ab	21.4 cde	0.970 bc	90.3 a
1/1-3	10.19 bc	336 ab	1.512 ab	19.9 de	1.010 abc	35.9 b
1/1-5	7.81 d	331 b	0.992 c	19.4 e	1.013 abc	23.3 c

Values followed by a different letter are significantly different (*t*-test, P ≤ 0.05).

As the submergence deepened and the duration increased, all treatment groups showed decreases in the aboveground dry weight and dry root weight per hole, adventitious root number, and longest root length; an exception was regarding the aboveground and underground dry weights and adventitious root number in the 1-d complete submergence group and the aboveground and underground dry weights and longest root length in the 1-d 1/2 group. One possible reason for this result is that submergence causes root hypoxia, which restricts aerobic respiration and promotes anaerobic respiration. As a result, the root system cannot gain sufficient energy, which impedes its normal growth and mineral absorption [32]; consequently, the roots shorten, and the root number and root weight decrease. Furthermore, Table 2 shows that the root width increased as the submergence deepened and the duration increased. In hygrophytes, oxygen easily diffuses into the soil while it is being transported toward the root tip [33], and sclerenchymatous cells normally form at the edge of the exodermis under hypoxic conditions (waterlogging) to stimulate the horizontal growth of rice roots, which minimizes oxygen loss from the roots into the soil [34]. Once this barricade of sclerenchymatous cells forms, it effectively weakens the radial outdiffusion of oxygen, enhances oxygen transportation toward the root tip, and thus promotes root growth in anaerobic environments.

In this study, the 1-d 1/1 group showed increases in the aboveground dry weight, dry root weight per hole and adventitious root number compared with the control group, which has not been reported previously. Presumably, although a short period (1 d) of complete submergence inhibits photosynthesis, it exerts more noticeable super-compensatory effects on the photosynthetic area and photosynthetic rate than 1-d partial submergence; thus, photosynthetic products display super-compensatory accumulation [30, 31].

### Effects of submergence during rice tillering on OS-ACS3 expression

Table 2 also shows that for Yangjing 4227 at the tillering stage in the 1/2, 2/3 and 1/1 groups, the expression of OS-ACS3 significantly increased compared with that of the control group after 1, 3 and 5 d of submergence. At different submergence depths, the expression of OS-ACS3 gradually decreased as the submergence duration increased. According to the literature [35, 36], under the stress of flooding, a low concentration of oxygen induces the synthesis of ACC in waterlogged rice to accelerate the production of C_2_H_4_, which promotes the continuous formation of aerating tissue close to the apical area and increases the activity of cellulase, reducing the diffusional resistance of oxygen toward the root system [37]. In addition, C_2_H_4_ stimulates the generation of adventitious roots and lenticel proliferation, which shortens the transmission distance of oxygen from the ventilation area to the root tip, promoting oxygen diffusion toward the root system. Furthermore, ethylene promotes the lignification and suberization of the root system, which decreases the radial leakage of oxygen in the roots and correspondingly increases the concentration of apical oxygen [38]. Therefore, the possible reasons for the findings of the present study are as follows. The oxygen concentration in the root tips of the treatment groups was lower than that of the control group, significantly increasing the expression of OS-ACS3. However, with prolongation of the waterlogging duration, the rice plants gradually developed adaptation mechanisms, with the oxygen concentration correspondingly gradually increasing and the expression of OS-ACS3 gradually decreasing.

As the submergence depth increased, the expression of OS-ACS3 in the 1/1 group was significantly higher than that in the 1/2 and 2/3 groups, especially after 1 d of submergence. The expression of OS-ACS3 in the roots of the 1/1 group was 5–7 times higher than that in the 1/2 and 2/3 groups. Specifically, the expression of OS-ACS3 in the plants with some parts above water (ventilated) was lower than that in the completely submerged (unventilated) plants, a finding that has not been reported in the literature. Presumably, the complete submergence led to a sudden change in the plants from an oxygen-rich state to a complete anoxic state (completely free of oxygen). This change in state sufficiently induced the expression of OS-ACS3 in the roots, which greatly promoted the synthesis of ACC synthase, the production of ACC, and thus the production of C_2_H_4_. However, the water layer blocked the leakage of C_2_H_4_, particularly in the 1/1 1-d treatment group, which greatly stimulated the stress mechanisms of the plants to cope with the waterlogging stress. The stress mechanisms stimulated some factors, such as the observed increases in photosynthetic rate and photosynthetically effective leaf area, to benefit plant growth rather than causing injury. In contrast, in the 1/2 and 2/3 groups, the parts above the submergence level were still in a normoxic state, and the oxygen concentration in the root tip was higher than that in the 1/1 group, resulting in reduced OS-ACS3 expression.

### Effect of submergence on the height of plants at tillering

**Fig 1:**
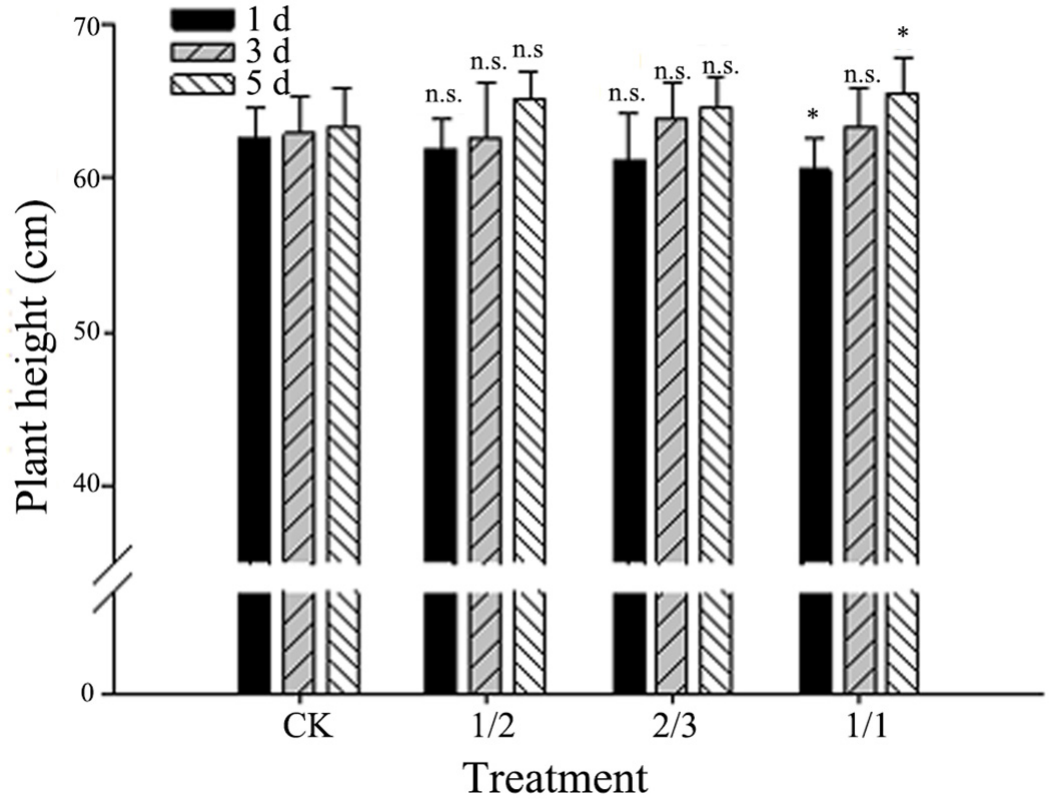
Effects of submergence on rice plant height at the tillering stage. The data were obtained at 1 d of post-waterlogging growth recovery. Compared with the control, the 1/1 group showed significantly decreased plant height after 1 d of submergence and significantly increased height after 5 d of submergence. The other treatment groups did not show significant differences. Each bar indicates the standard error (n = 5). CK, control group; * P < 0.05 vs. the control; and n.s., not significant vs. the control (*t*-test).

The height of the plants at tillering measured on the second day after the different submergence durations is shown in Fig 1. In the 1/2 and 2/3 groups, the heights of the plants did not significantly increase or decrease compared with the control group after 1, 3, and 5 d of submergence. Conversely, in the 1/1 group, the height did significantly increase and significantly decrease after 1 and 5 d of submergence, respectively. At the different submergence depths, 1 d of submergence decreased or significantly decreased the height of the plant, and 5 d of submergence increased or significantly increased the height of the plant; 3 d of submergence did not cause significant changes. Our results are consistent with those reported in the literature [3, 5, 7, 36] and may indicate that the increase in plant height becomes more noticeable with prolonged submergence. The underlying reason is presumably as follows. On the one hand, rice acquires oxygen by extending its stem and leaves above the water level after waterlogging to avoid hypoxia [39]; on the other hand, waterlogging promotes the production of “stress ethylene”, which interacts with growth hormones to promote cell elongation; however, such growth is not classified as normal growth because it is not caused by an increase in cell number [36].

### Effect of submergence during tillering on nitrogen and phosphorus content

The effect of submergence on nitrogen and phosphorus content in rice at the tillering stage is summarized in Table 3. In the 1/2 group, the nitrogen and phosphorus contents decreased after different submergence durations compared with the control group. However, the level of accumulated nitrogen after 1 d of submergence was slightly increased, whereas the levels of accumulated nitrogen and phosphorus after the other treatments were notably decreased. In the 2/3 group, the nitrogen content after 1 d of submergence decreased by 11.5% but increased by 3.0% and 3.2% on days 3 and 5, respectively, compared with the control group. The accumulated nitrogen, the phosphorus content, and the accumulated phosphorus all decreased after the different submergence durations. In the 1/1 group, the nitrogen content significantly increased by 16.6%, 35.4%, and 14.3%, and the accumulated nitrogen significantly increased by 17.4% and 21.6% but significantly decreased by 30.4% after 1, 3, and 5 d of submergence, respectively, compared with the control group; the phosphorus content and accumulated phosphorus were significantly decreased compared with the control.

**Table 3:** Effects of waterlogging in the tillering stage on nitrogen and phosphorus content and accumulation in rice plants examined at 7 d of post-waterlogging recovery growth.

Submergence depth- duration (d)	Nitrogen content (%)	Phosphorus content (%)	Nitrogen accumulation (g/hole)	Phosphorus accumulation (g/hole)
Control-1	2.246 cd	0.895 a	0.234 c	0.093 a
Control-3	2.123 defgh	0.827 b	0.241 c	0.094 a
Control-5	2.070 efgh	0.743 c	0.265 b	0.095 a
1/2-1	2.152 def	0.815 b	0.241 c	0.091 ab
1/2-3	1.966 h	0.741 c	0.212 d	0.080 cd
1/2-5	1.993 fgh	0.737 c	0.210 d	0.078 d
2/3-1	1.988 gh	0.848 ab	0.201 d	0.086 bc
2/3-3	2.186 de	0.711 cd	0.212 d	0.069 e
2/3-5	2.136 defg	0.712 cd	0.177 e	0.059 f
1/1-1	2.618 b	0.861 ab	0.275 b	0.090 ab
1/1-3	2.873 a	0.803 b	0.293 a	0.082 cd
1/1-5	2.365 c	0.663 d	0.185 e	0.052 g

Values followed by a different letter are significantly different (P ≤ 0.05, t-test).

Correlation analysis revealed a significant positive correlation between the panicle number and the nitrogen content, with a correlation coefficient of 0.7328; however, the correlation coefficient between panicle number and phosphorus content was -0.3606 (n = 12), which was not significant.

Long-term waterlogging at the tillering stage can decrease the phosphorus content in rice plants. Because phosphorus is an element that can be reused by the plant, a decrease in phosphorus content influences middle and late plant growth, particularly during the rice flowering and filling stages. At these stages, the requirement for phosphorus reaches a peak that is associated with vigorous photosynthesis and carbohydrate metabolism, and a lack of this element can significantly decrease rice yield [40]. This may be an important reason for the large decrease in rice yield after long-term waterlogging.

The findings of this study show that 1 d of complete submergence significantly increased the yield of rice, which contradicts the assumption that a short-term waterlogging disaster is detrimental to rice growth [3–9]. This discrepancy may be partially due to the differences between this study and previous studies in terms of the duration of rice submergence, submergence temperature, submergence stage, and rice cultivar selected.

Based on the above analysis, the reason for a significant increase in rice yield after 1 d of complete submergence can be summarized as follows. First, 1 d of complete submergence significantly increases the efficient leaf area of the plants without significantly influencing their photosynthetic rate. These effects promote the production and accumulation of photosynthate as well as grain filling [30, 31]. Second, 1 d of complete submergence significantly increases the nitrogen content and accumulated nitrogen in rice, which positively correlates with panicle number. Third, 1 d of complete submergence decreases the height of the plants, which means that they do not consume a large quantity of carbohydrates for increasing their height, thereby enhancing energy accumulation [41].

This study has some limitations. First, the water used in this study was static, clean, tap water. Muddy water and changes in the flow rate may produce different results. Second, this study could not completely simulate actual field waterlogging conditions. Therefore, whether the effects of waterlogging at the rice tillering stage on rice growth and yield observed in this study are applicable to actual fields remains to be explored. Third, the allowable water storage control duration at the rice tillering stage proposed in this study needs to be verified in large-size field practice. Fourth, this study is only concerned with changes in rice after being faced with different waterlogging depths and durations during the tillering stage. Further studies on changes in rice at other growth stages and the effects of different waterlogging seasons and temperatures remain to be conducted.

## Conclusions

Based on the results obtained in this study, the following conclusions can be drawn:

1. The decrease in rice yield can be controlled to 10% after the plants suffer from half, 2/3, and complete submergence for 1–3 d of field water storage;
2. It is possible that a certain degree of water storage control during the rice tillering stage does not cause serious yield loss and that the space for water control in rice fields can be expanded.

The results of this study may be of great practical significance for increasing rainwater storage depth, expanding the water control space in paddy fields, increasing rainwater utilization efficiency, conserving irrigation water, and reducing labor investment.
